# Considerations for and Mechanisms of Adjunct Therapy in COPD

**DOI:** 10.3390/jcm10061225

**Published:** 2021-03-16

**Authors:** Rachana Mandru, Christine Y. Zhou, Rachel Pauley, Robert M. Burkes

**Affiliations:** 1 Division of Pulmonary, Critical Care, and Sleep Medicine, University of Cincinnati, Cincinnati, OH 45221, USA; mandrura@ucmail.uc.edu; 2 Department of Internal Medicine, University of Cincinnati, Cincinnati, OH 45221, USA; zhouct@ucmail.uc.edu (C.Y.Z.); pauleyrl@ucmail.uc.edu (R.P.)

**Keywords:** COPD, roflumilast, azithromycin, vitamin D, hypertonic saline, n-acetylcysteine

## Abstract

Inhaled bronchodilators and corticosteroids, when indicated, form the backbone of COPD therapy. However, over the last decade there has been an emergence of adjunct therapies in oral or inhaled form that are now part of the therapeutic approach to COPD. While these therapies have shown to be beneficial when used in the appropriate instances, there are particular considerations that need to be minded when using these therapies. This review article discussed the mechanism of roflumilast, macrolide antibiotics, other chronic antibiotic regimens, vitamin D supplementation, oral corticosteroids, n-acetylcysteine, and nebulized hypertonic saline, the clinical data behind each of these therapies, adverse events associated with therapy, and the expert recommendations for their utilization. Our goal is to provide a brief but informative and clinically useful review of commonly encountered therapies used in advanced COPD.

## 1. Introduction

Chronic obstructive pulmonary disease (COPD) is a preventable, progressive, potentially debilitating, but treatable disease which is common worldwide [1]. COPD is marked by lung function loss, worsening functional status, and exacerbations leading to the need for more intensive therapies and/or healthcare utilization. The Global Initiative for Chronic Obstructive Lung Disease (GOLD) has produced guidelines for the treatment of patients with COPD including inhaled bronchodilators and corticosteroids, as well as other, adjunct therapies, thought to potentially have some benefit in COPD. This article will provide a review of ‘add on’ therapies in the treatment of COPD and prevention of poor COPD-related outcomes and include benefits, risks, and special considerations for each therapy.

It should be noted that COPD is often complicated by a complex interface of COPD signs, symptoms, and physiologic derangements with other comorbidities. The GOLD guidelines [1] suggest assessment of concomitant comorbid diseases and having these addressed as there is a notable overlap in symptomatic manifestations and need for healthcare utilization among commonly comorbid conditions to COPD (heart failure, coronary artery disease, obstructive sleep apnea, diabetes mellitus, obesity, etc.). Guidelines are also clear that those with COPD, no matter the severity, also benefit from preventative measures that will have a notable effect on disease course including vaccinations, smoking cessation, and the need for pulmonary rehabilitation. Alpha-1-antitrypsin disease should be screened in all patients with airflow limitation [2] and longitudinal care for this disease and therapeutic approach is of the utmost importance and deserving of its own review.

Prior to discussing adjunct therapies, it is important to note that triple therapy with dual bronchodilators and an inhaled corticosteroid is an important medication regimen in COPD care [3]. This regimen has been associated with lower hazard rates of death from pulmonary or cardiovascular causes in one study when compared to dual bronchodilators alone [4], but this relationship may only be seen in those on high-dose inhaled corticosteroids [5]. It will be pointed out that the following additive medications which are recommended by guidelines^1^ (macrolides and roflumilast) are indicated in persons already on maximal inhaled therapy.

## 2. Roflumilast

Airway inflammation and oxidative stress are the key processes contributing to the symptom burden and recurrent exacerbations in patients with COPD. Roflumilast is an FDA approved novel therapy for COPD which acts by inhibiting phosphodiesterase-4 (PDE4), a major metabolizing enzyme that is primarily found in the lung tissue [6]. It selectively inhibits hydrolysis of cyclic adenosine monophosphate (cAMP) in the inflammatory cells of the lung compartment [7]. The inhibition of cAMP breakdown has several positive knock-on effects preventing bronchospasm in COPD patients including decreased release of pro-inflammatory cytokines and mediators, reduced cellular apoptosis, and inhibition of chemotactic mechanisms promoting inflammation [6,8]. Due to these putative benefits, current Global Initiative for Chronic Obstructive Pulmonary Disease guidelines suggest that patients with ‘severe’ COPD (FEV1%-predicted < 50%), symptoms of chronic bronchitis, and a history of exacerbations not fully controlled on a maximal inhaled medication regimen may benefit from the addition of roflumilast to their medication regimen in an attempt to prevent exacerbations, and has been suggested as a potential steroid sparing agent [1,6]. Further, the GOLD guidelines suggest that those with persistent exacerbations despite maximal medical therapy and peripheral eosinophil counts less than 100/mcL (however, emerging data suggests the peripheral eosinophil recommendation should be abandoned [9,10]) may also benefit for the administration of roflumilast to their therapy regimen. As such, it is important to understand the literature surrounding the clinical effectiveness of roflumilast.

Clinical efficacy of Roflumilast has been evaluated in multiple studies in a well-defined patient population. In 2015, a randomized, double-blind trial by Martinez et al. was published in *The Lancet* comparing the administration of roflumilast to placebo [11]. This study included participants with post-bronchodilator FEV1 ≤ 50%, chronic bronchitis symptoms, and at least two exacerbations in the year prior to enrollments. Participants were randomized in a 1:1 fashion to receive 500 μg Roflumilast vs. placebo for 52 weeks. Participants in this study were all on LABA/ICS combinations and were allowed LAMA treatment in the background. In the intention to treat group, the rate of moderate to severe exacerbations was 14.2% lower in the treatment group with a (RR of 0.858 [95% CI 0.740–0.995]; *p* = 0.0424). In this study the most frequently reported adverse events over one-year follow-up were diarrhea (15% in treatment vs. 19% in placebo), pneumonia (4% in treatment vs. 5% in placebo), and COPD exacerbation (15% in treatment vs. 19% in placebo).

The accompanying RE(2)SPOND trial [12] was performed with an analogous study cohort and endpoint of total reduction in exacerbations. The reduction in any exacerbations per year was reduced 8.5% (95% CI 0.81–1.04; *p* = 0.16). This endpoint did not meet statistical significance, however, post hoc analyses were interesting in that those with at least one hospitalization in the year prior to enrollment saw a 21% lower risk of having an exacerbation that led to need for outpatient therapy during follow-up. This study also described a larger difference in adverse event drop-out rate for treatment arm (11.7% vs. 5.4%) then did REACT. Further studies showed starting roflumilast on inpatients with COPD exacerbations did not affect 30-day rehospitalization rates [13].

The GOLD criteria suggest that roflumilast is a reasonable addition to the mediation regimen of patients who are currently on an inhaled corticosteroid, have severe COPD, have previous COPD exacerbations, and complain of symptoms of chronic bronchitis or have persistent exacerbations despite maximal inhaled therapy and a low peripheral eosinophil count [1]. The beneficial effect may be stronger in those with multiple exacerbations in a year and/or have been hospitalized in the year prior. Special attention needs to be paid to gastrointestinal side effects as a cause of non-compliance and caution should be used in underweight patients as well as patients who suffer from depression should be monitored for worsening mood [1,14]. A summary of recommendations for the use of roflumilast and all other agents are discussed in Table 1.

## 3. Macrolides

Recently the role of macrolides in the prevention of COPD exacerbations has become of interest and the utilization of macrolide antibiotics for COPD prevention has seen wide clinical utility. Macrolide antibiotics, when taken chronically, have several effects on immunological activity in the lungs [15]. First, macrolides exert anti-inflammatory action by lessening the local immune response to noxious agents [15,16]. Macrolides have also been suggested to reduce the production of the highly viscous MUC5AC by airways cells [17] and to promote the production of antimicrobial peptides which are an important first-line defense of the airways and which modulate the downstream immune response [18]. Lastly, macrolides can decrease or interrupt the biofilm formation of colonizing pathogens inhibiting the hyperinflammatory, deleterious feed forward cycle of chronic infection and airways disease progression [19]. Studies cataloguing the mechanism of the anti-inflammatory effect of macrolides by measuring various biomarkers of inflammation have reported that persons taking macrolides have lower sputum neutrophils and also show decreases in the peripheral concentration of cytokines of both the Th1 and Th2 pathways [20]. Trial data suggests these measured biological changes also have clinical significance.

A prospective, double-blind study conducted by Albert et al. assessed the median time for exacerbation in patients receiving 250 mg of azithromycin daily for 12 months vs. placebo in participants with COPD, at least 10 pack-years of smoking, who had either received systemic glucocorticoids or had presented for inpatient care in the year prior to enrollment. The median time to exacerbation was 266 days in treatment group (95% CI 277–313 days) vs. 174 days in the placebo group (95% CI 143–215 days) with a *p* value < 0.001. Subset analysis of this study suggests that these findings were driven by fewer office visits along with fewer emergency room (but not hospitalizations) in the treatment arm. Number needed to treat [NNT] to prevent one acute exacerbation was 3. It is also important to note that of the 1142 included in the study 870 were ex-smokers. Current smoking status was equally distributed as 21% of the treatment arm and 23% of the placebo arm were smokers at the time of enrollment. Further, there was a significant increase in audiogram-detected hearing decrement in the treatment arm (27% vs. 21%), with the majority of participants regaining their hearing after azithromycin was discontinued. There were no differences in deaths due to cardiovascular events over the course of this study. Finally, the emergence of macrolide-resistant colonizing organisms was observed over the course of the study in the treatment arm, potentially complicating treatment of future COPD exacerbations. 

Han et al. [21] using the same cohort as above, performed a post hoc analysis to understand which COPD patients may gain the most benefit from the administration of azithromycin for the prevention of COPD exacerbation. Their analysis showed ex-smokers, older persons, and those with milder disease were more likely to glean benefit from the addition of azithromycin to their treatment regimen. Interestingly, there was no difference in the response based on inhaled medication usage, sex, or chronic hypoxic respiratory failure requiring supplemental oxygen.

These findings have been noted in other smaller trials of azithromycin and erythromycin as has been reported in meta-analysis of 12 studies [22]. The Mansel–Hanzel odds ratio in these 12 studies comparing treatments (which was variable in individual studies for both agent and dosing frequency) showed a 60% decrease in odds of COPD exacerbation over a 12 month time horizon. Further sub-analyses show that there was no difference (but a smaller sample size) between treatment and placebo at three- or six-months and the effect of clarithromycin is numerically larger than with azithromycin, while the associations between clarithromycin and COPD exacerbation prevention were null. Please note, there was a high degree of heterogeneity (I^2^ =68% in the main models) in the studies included in the meta-analysis. At the time of this writing the GOLD guidelines [1] do not favor azithromycin over erythromycin nor do they specify dosing strategy, and comfort with an agent and dosing strategy should be made on a patient-by-patient basis. 

The addition of azithromycin is also recommended as a potential add on medication for the prevention of COPD exacerbations per the GOLD guidelines [1]. The target population are non-smoking individuals with a history of exacerbations on maximal medical therapy, especially those with eosinophil counts less than 100/mcL. While the effectiveness seems to not be limited to those on certain drugs, the best responses to this medication are best seen in former smokers and those with milder disease and there may be a benefit in older persons. Additionally, it is unknown if there is benefit after one year of therapy. Side effects need to be considered before starting this medication and during therapy which include hearing loss, the emergence of macrolide resistant organisms, as well as prolonging the QT segment although this potentially fatal side-effect was not observed in high frequency in studies. Please see Table 1 for agent doses and indications.

## 4. Antibiotics Directed at Chronic Bacterial Colonization of the Airways

Chronic airways infection is a complicating factor in the progression of COPD. Impaired host defenses and structural damage to airways allow for chronic colonization with bacteria which promotes a hyperinflammatory state leading to disease progression [23]. chronic bacterial colonization in COPD is thought to lead to more rapid lung function loss, increased exacerbation events, and a worse health related quality of life [24]. These concerns have formed the basis of study into the use of pulsed or chronic antibiotics in the treatment of COPD symptoms and the prevention of COPD exacerbations. While theoretical benefit from antibiotics directed at chronic colonizers is attractive, at the time of this writing the GOLD guidelines do no recommend chronic antibiotics directed at chronic airway infection [1]. 

A relatively large trial (*n* = 573 in the treatment arm; *n* = 584 in the placebo arm) of stable participants with COPD was conducted to determine if a single dose of moxifloxacin administered every five days for eight weeks could lead to a reduction in odds of exacerbation over 48 weeks of study [25]. In the intention to treat arm, there was a not-statistically significant 29% decrease in the odds of exacerbations over 48 weeks in the treatment arm (95% CI 0.65–1.01; *p* = 0.06). In the per protocol arm there was a lower odds of exacerbation event in the treatment group which was associated with an improved quality of life. The differential findings between per-protocol and intention to treat arms suggest that there is an inherent issue with this therapy and approach that may make the intermittent dosing difficult to adhere to or that the long-term side effects of moxifloxacin may not be tolerable. A Cochrane Review [26] incorporating other smaller studies did not show evidence of benefit from the administration of chronic antibiotics in the prevention of COPD exacerbations when the above study was pooled with smaller studies with similar methodology. 

Inhaled antibiotics for the prevention of exacerbations are attractive agents for the prevention of COPD exacerbations due to the rationale that the inhaled method of delivery will direct drug directly to the infected airway rather than exposing a patient to the side effects of systemic medication. This approach forms the backbone of exacerbation prevention in cystic fibrosis [27], but the data in COPD, while promising, has yet to be recommended by the GOLD guidelines [1]. One small study of 36 participants was performed to assess the benefit of inhaled colistin in prevention of COPD exacerbations. Pre/Post assessment of exacerbations show a >100% decrease in exacerbations over one year. This finding was supported by retrospective analysis showing patients on inhaled colistin for at least three months had fewer exacerbations of COPD [28]. Another small study (*n* = 13) evaluated the use of inhaled tobramycin in patients with COPD and *Pseudomonas* colonization [29]. Levels of hyperinflammatory cytokines and *Pseudomonas* load were decreased, but clinical outcomes were not reported in this trial. Caution needs to be exercised when considering the outlook inhaled antibiotics for the prevention of poor COPD outcomes as there is a need for these studies to be validated in larger cohorts and in a randomized, placebo controlled, double-blinded trial with a robust means to validate patient reported outcomes. At this time, these are not recommended by GOLD guidelines [1].

## 5. Mucolytics

Oxidative stress leads cellular damage in COPD via inflammatory pathways involving nuclear factor kappa B, TNF-alpha, interleukin 8 as well as damage to intrinsic anti-inflammatory pathways [30,31]. Interest in treating COPD with N-acetylcysteine (NAC) is based on the premise that a reduction in oxidative stress will reduce inflammation and act as a mucolytic, mitigating the hyperinflammatory state that leads to disease progression. Inhaled NAC acts as a mucolytic by hydrolyzing bonds between mucin glycoproteins, which reduces the viscosity of mucous in vitro [32,33]. NAC can be administered either orally or via nebulization in COPD patients. The administration of oral NAC is simpler, however when taken orally, NAC is rapidly metabolized and has not been found to become present in bronchoalveolar lavage fluid [34]. Further, the mechanism of action when administered by other means is hypothesized to be due to the reduction of oxidative stress rather than direct mucolysis [35]. 

Both inhaled and oral NAC have been studied as potential agents to relieve symptoms and modify disease progression in COPD. In a multi-center, intention-to-treat study of daily oral NAC (600 mg daily) versus placebo [36], NAC was not found to significantly impact lung function decline or exacerbation rate over one year of follow-up. The PANTHEON trial [37] investigated high dose NAC (600 mg NAC twice daily) for moderate to severe COPD as defined by pulmonary function testing. Participants randomized to twice daily NAC, as opposed to placebo, had significantly fewer COPD exacerbations in the one-year trial period (1.16 exacerbations per pt year vs. 1.49, *p* = 0.0011). Strength of this data is limited by subjective definition of COPD exacerbation and lack of data regarding severity of COPD exacerbation. Literature regarding the efficacy of inhaled NAC for muco-ciliary clearance in COPD is sparse. A 2020 Cochrane review of 38 trials of mucolytics for COPD (21 studies) [38] (2), concluded that there may be quality of life and disease modifying benefit from mucolytic use in COPD. However, the review concluded that evidence of reduction in mortality or morbidity is lacking. 

Use of oral and inhaled NAC and other mucolytics remains an area where further study is needed. Side effects are generally thought to be mild and mostly are from gastrointestinal upset and the notably unpalatable taste of oral NAC [1]. The mucolytic, carbocysteine has shown similar results to NAC [1]. Additionally, while a Cochrane Review on the subject concludes there may be benefit to mucolytic agents (NAC and others), there is large heterogeneity in studies and the results are driven by older studies (prior to 1990) and massive advancements have been made in COPD drug therapy since then [39]. Further, as stated above, the mechanism in vitro is nebulous and oral NAC does not seem to enter the lung compartment. The most recent GOLD guidelines indicate possible benefit from NAC and other mucolytic therapy as an adjunct treatment in COPD, however the optimal patient population and dose remain unclear at the time of this writing and no formal recommendations have been made for employing this therapy in the care of COPD patients [1]. 

## 6. Nebulized Hypertonic Saline

Nebulized hypertonic saline (HTS) is often used clinically to assist in sputum expectoration and has thus been explored as a therapeutic modality in patients with chronic lung disease. While there is evidence of clinical benefit in patients with cystic fibrosis (CF) [40,41], there is limited data in patients with COPD. The exact mechanism of action of HTS remains unclear, but current understanding points to a multimodal effect. HTS creates an endobronchial osmotic gradient, leading to increased passive secretion of water into the airways through apical aquaporin channels [42], while also stimulating airway neurons to trigger epithelial secretion of ASL [43]. HTS also has mucolytic properties which decrease mucous viscosity, allowing for the reformation of mucous as it ascends the bronchial tree and promoting overall clearance. Lastly, HTS triggers cough, providing further mechanical clearance [44,45]. 

Data on clinical outcomes in patients with COPD undergoing HTS therapy is limited and results are mixed. Bennett et al. [46] conducted a trial comparing HTS to normal saline in 22 patients with chronic bronchitis and found no improvements in spirometry, symptoms, or mucus clearance after 2 weeks of therapy. Mucus clearance was actually reduced after therapy in central airways. A randomized controlled trial in 68 patients with COPD combining HTS with an 8-week exercise program showed improvement in 6-min walk distance. However, the effect was greater in the isotonic saline group compared to the HTS group. There was symptomatic improvement with both groups. Unfortunately, this study was unable to differentiate if these effects were a result of nebulized saline therapy or the exercise program [47]. 

HTS therapy appears safe in patients with COPD. However, there is a transient acute fall in forced expiratory volume in 1 s (FEV1) in many patients despite pre-treatment with a beta-agonist. In a study of 20 patients [47] with COPD, a challenge of HTS had to be terminated in all but 2 patients due to a >20% fall in FEV1, and all patients displayed a 20% fall in forced inspiratory volume in 1 s. Despite the perceived safety of HTS, there is little demonstrated clinical benefit at this time of utilizing this therapy in COPD, either for symptoms or to treat exacerbations. Further study in this field should focus on potential COPD subtypes (COPD-bronchiectasis overlap) that may particularly benefit from this intervention. At the time of this writing, this therapy is not indicated for management of COPD alone.

## 7. Vitamin D

Supplementation of vitamin D is an attractive additive therapy for those with COPD who are vitamin D deficient for a variety of reasons. 1,25-Hydroxy-vitamin D, the active form of the vitamin, has a pleitropic effect on the immune system but promoting the expression of genes coding for innate defensins, regulating macrophage differentiation, signaling cell apoptosis, modulating cellular adhesion, and promoting a more anti-inflammatory response to invading pathogens and noxious stimulants [48,49,50,51]. Further, there is a putative musculoskeletal benefit to supplementation of vitamin D in pulmonary rehabilitation patients [52]. At the current time, researchers are attempting to more accurately define the clinical benefit of vitamin D sufficiency and supplantation in the COPD population.

Observational studies suggest persons with COPD and 25-OH-vitamin D levels < 20 ng/mL may lose more lung function over time and report higher incidence of exacerbations [53], however, this study could not determine if supplementation of those with vitamin D deficiency was clinically meaningful. Trials assessing the effect of vitamin D supplementation in deficient participants have been performed and, taken as single studies, it does not seem vitamin D supplementation has much benefit in protection against COPD exacerbations and other longitudinal outcomes [54,55]. There are several nuances of these studies that suggests the role of vitamin D supplementation in COPD requires further investigation, however, with the major one being that participants in both of these trials started their supplementation with vitamin D levels very near normal on average. Joliffe [56] conducted a meta-analysis with the two abovementioned trials as well as a third [57], and concluded that those who have very low vitamin D levels (<10 ng/mL or <25 nmol/L) have a significantly reduced incidence rate of COPD exacerbations if supplemented with vitamin D. Likely because of this compiled data, GOLD now recommends considering vitamin D supplementation in deficient COPD patients [1]. It should be noted that patients should have vitamin D levels and deficiency addressed by primary care providers or endocrinologists and at this time no solid recommendations can be made by guidelines as to who would best benefit from a purely pulmonary standpoint from vitamin D supplementation. 

## 8. Oral Corticosteroids in COPD

Oral glucocorticoids are a mainstay of therapy in COPD exacerbations [1]. Due to its wide range of anti-inflammatory effects and its titratability to a patient-directed dose, oral steroids may seem to have potential benefit in stable-but-severe outpatient COPD. However, the use of oral glucocorticoids as chronic maintenance therapy in COPD is highly contested. Currently, the GOLD guidelines do not recommend their use in chronic daily therapy [1]. Despite this, it is not uncommon for those with advanced COPD to be placed on daily glucocorticoids. Due to this, we will review the literature surrounding the use of these agents.

Oral glucocorticoids have been investigated for several decades as a possible disease modifying agent for COPD, however data supporting their efficacy remains sparse. Postma et al. [58] published a study in 1988 in 139 patients with chronic airflow obstruction that concluded that doses of oral prednisolone of at least 10 mg a day could slow progression of obstruction. This study did not differentiate between asthma and COPD, limiting its application to COPD treatment. A 1991 meta-analysis by Callahan et al. [59] reported that patients with stable COPD treated with oral glucocorticoids had a 20% or more increase in baseline FEV1. However, the strength of this finding was limited by duration of treatment: only 1 of the 10 studies that met criteria evaluated a glucocorticoid course of 8 weeks, with all other studies using courses of 14 days or less. In 1996, Renkema et al. [60] published a parallel group study of 58 non-allergic patients evaluating the use of inhaled budesonide with or without oral prednisolone 5 mg over 2 years. This study found no significant difference in FEV1 decline between the groups. A Cochrane review [61] published in 2005 reported no significant difference in FEV1 or health related quality of life with prolonged oral glucocorticoid use, but was only able to include 2 studies, one of which was the previously mentioned study by Renkema et al. It is difficult to draw any strong conclusions from these studies, although the available data appears to show a lack of clinical benefit from long term oral glucocorticoids. 

Long term steroid use comes with significant concerns. In cohort of 556 patients with COPD in the Netherlands [62], oral glucocorticoids at doses of >10 mg for at least 6 months was associated with a dose-dependent increase in mortality without significant difference in FEV1. Another multi-center cohort study [63] of 444 patients with COPD that used propensity-score-matching, long term oral glucocorticoid use for more than 6 months was associated with increased mortality, with a hazard ratio ranging from 1.50 to 1.73. Glucocorticoids bear significant risk including increased mortality, muscle wasting, hyperglycemia, osteoporosis, and other effects [12]. These adverse effects are well documented in the literature.

The initiation of long-term glucocorticoid use is generally considered in patients who are not controlled on other therapy as a ‘salvage’ medication. The balance of data does not suggest that clinicians should regularly prescribe oral glucocorticoids and if this selection is made, the lowest possible dose of these agents is preferable. Further, for those patients currently on chronic oral glucocorticoid therapy, discontinuation has been shown to be safe and should be considered [64]. 

## 9. Conclusions

Several therapies outside of inhalers exist in the preventative treatment of COPD. However, there special considerations need to be paid before selecting an add-on therapy. We have described the background evidence of some commonly used therapies and explained clinical situations where these therapies may be beneficial. We also believe by presenting the data behind these treatments and the pitfalls of each approach, we illustrate the need for continued research into new, targeted therapies and the specific target population of patients that will benefit from adjunct therapy. At this moment, we recommend making all therapeutic decisions in COPD based on individual patients and in concert with GOLD or other association guidelines.

## Figures and Tables

**Table 1:** Available adjunct therapies in COPD and GOLD 2021 [1] recommendations.

Agents	Dosing	Indications	Side Effects
Roflumilast	500 mcg orally daily 250 mcg orally daily for 4 weeks followed by 500 mcg daily	While on maximal inhaled therapy to prevent exacerbations: Chronic Bronchitis FEV1/FCV < 0.50 History of AECOPD or History of AECOPD Eos < 100/mcL	Weight loss Sleep disturbance Abdominal pain Worsening mood disorder
Macrolides	Azithromycin 250 mg daily Azithromycin 500 mg three times weekly Erythromycin 250 mg twice daily	While on maximal inhaled therapy to prevent exacerbations: Non-smoker History of AECOPD or History of AECOPD Eos <100/mcL	Hearing loss QTc prolongation Gastrointestinal upset Development of resistant organisms
Mucolytics	Various inhaled formulations 600 mg oral daily (NAC) 375 mg oral, two capsules, three times daily (carbocysteine)	Potential benefit but there is no target population at this time	Bad taste Bronchospasm Nausea Pruritis
Prophylactic antibiotics	Moxifloxacin 400 mg oral daily for 5 days every 8 weeks Inhaled colistin 75 mg nebulized every 12 h Inhaled tobramycin 300 mg nebulized every 12 h	These are not indicated at the time of this writing but inhaled agents hold promise and should be studied further	Side effects per select agent. Agents should be cycled on and off therapeutic plan to prevent side effects
Hypertonic saline	7% or 3% vials nebulized twice daily	Not recommended at this time	Bronchospasm Increased coughing
Vitamin D	50,000 units orally weekly for 8 weeks in those with 25-OH-vitamin D serum levels < 20 ng/mL	May have benefit in those with vitamin D deficiency at preventing AECOPD	Hypercalciuria Hypervitaminosis D Hypercalcemia Anorexia Nephrotoxicity
Oral Corticosteroids	Dose variable	Not recommended	Weight gain Osteroporosis Diabetes Adrenal insufficiency Immunosuppression Infection Myopathy Etc.

Abbreviations: NAC: N-acetylcysteine; OH: Hydroxy.

## Data Availability

There is no new data generated by this review. The data for the respective studies discussed is available prt the data sharing plan of each study.
